# Hydrothermal Synthesis of a Valence State Constant High-Entropy Perovskite Sr(TiZrHfVNb)O_3_ with Improved Photoresponsiveness

**DOI:** 10.3390/ma17174275

**Published:** 2024-08-29

**Authors:** Yihua Bai, Ke Gan, Xiaohu Li, Dongping Duan

**Affiliations:** 1 School of Materials Science and Engineering, University of Science and Technology Beijing, Beijing 100083, China; u202140611@xs.ustb.edu.cn; 2 CAS Key Laboratory of Green Process and Engineering, National Engineering Research Center of Green Recycling for Strategic Metal Resources, Institute of Process Engineering, Chinese Academy of Sciences, Beijing 100190, China; 3 University of Chinese Academy of Sciences, Beijing 100049, China; 4 Institute for Advanced Materials and Technology, University of Science and Technology Beijing, Beijing 100083, China; 13051691588@sina.cn

**Keywords:** high-entropy oxides, perovskite, hydrothermal process, ionic valence states, photoresponsive

## Abstract

A vanadium ion valence state constant high-entropy perovskite system was synthesized using the hydrothermal method with a trivalent vanadium ion as the vanadium source. The B-site of the perovskite crystal lattice was loaded with five atoms in equal proportions. We tried to synthesize the Sr(TiZrHfVNb)O_3_ high-entropy system using different methods. However, the valence state of the vanadium ion could only be kept constant using the hydrothermal process in the valence balanced high-entropy composition system. There was significant vanadium element segregation and second phase in the Sr(TiZrHfVNb)O_3_ system prepared using the solid-state reaction process. Also, obvious vanadium ion valence state ascending from V^3+^ to V^5+^ appeared in this high-entropy system with an increase in calcination temperature. Inconspicuous vanadium element segregation appeared at 900 °C, the significant segregation phenomenon and second phase appeared at 1200 °C, and the particle size increased with the temperature. This meant that the high-entropy value could not only stabilize the crystal phase, but also stabilize the ionic valence state. Moreover, the constant trivalent vanadium ion valence state could provide coordinated performance with a wide optical response range and a low band gap for the high-entropy system. This suggests that the system might grow a potential ceramic material for optical applications.

## 1. Introduction

Since the first high-entropy oxide rock salt structure (MgCoNiCuZn)O was reported by Maria in 2015 [1], high-entropy ceramics (HECs) have gained much attention due to their special properties [2,3,4]. HECs are solid solutions of inorganic compounds with one or more Wyckoff sites shared by equal or near-equal atomic ratios of multi-principal elements [5]. Many kinds of HECs have already been prepared, such as rock salt [6,7], fluorite [8,9], spinel [10,11,12,13], and so on.

Perovskite is a simple-structure crystal that has a high tolerance factor [14]. It is widely researched as a photoelectric material for photovoltaic devices, photo catalysis, and so on [15]. Inorganic perovskite (SrTiO_3_, BaTiO_3_, etc.) has its own advantages such as high stability and high-temperature resistance [16]. Therefore, it is one of the most popular entropy-engineered ceramics [17]. Usually, bandgap widths of inorganic perovskite are often greater than 3 eV, resulting in a response in the UV wavelength range only [18]. Many investigations have improved the light absorption range using element doping, but this is not ideal in practical applications [19]. The high-entropy component design can reconstruct the electronic structure and modulate the band gap, which is a feasible way to broaden the light absorption range of perovskite materials. For entropy engineering of perovskite, the atomic radius of the design element can be selected in a large range. Even if cube perovskite fails to form, it can be distorted into tetragonal or quadrature phase structures.

So far, the conventional preparation methods for high-entropy ceramic perovskite oxides include the solid-state reaction [20,21], sol-gel [22,23], or co-precipitation processing [24]. These methods tend to make the valance states of some elements change during the reaction, such as vanadium, which disorganizes the material structure [25]. In contrast, a hydrothermal reaction provides an approach to obtain a sample at a lower temperature. Vanadium-based materials are common optical function materials such as SrVO_3_, Sr_3_(VO_4_)_2_, and SrV_6_O_11_, etc. [26]. Previous researchers have already synthesized a few high-entropy systems with vanadium elements [27,28]. The introduction of a vanadium element not only effectively inhibits grain growth and improves the mechanical properties [29], but also eliminates Nb segregation and significantly improves the densification process [30]. However, the valence of vanadium is hard to control, which is detrimental to the preparation of HECs.

In the present work, we design a high perovskite system Sr(TiZrHfVNb)O_3_. The hydrothermal process is employed to synthesize this system to improve the photoresponsiveness of perovskite. A somewhat surprising experimental result is that this system could only be synthesized using the hydrothermal method. The most commonly used ceramic powder calcination process cannot achieve the entropy stability of the perovskite phase. This advances our understanding of the relationship between the entropy stability of the phase structure and the ionic valence state of the designed high-entropy elements.

## 2. Experimental Section

### 2.1. Materials and Preparation

For the synthesis of high-entropy ceramic powder using the one-step hydrothermal method, liquid raw material TiCl_4_ and powder raw materials ZrCl_4_, HfCl_4_, VCl_3_, FeCl_3_, NbCl_5_ were purchased from Shanghai Macklin Biochemical Technology Co., Ltd. (Shanghai, China). These chlorides with the most stable valence state of the elements were used as their source, except for vanadium(III) trichloride which was used as vanadium source. SrCl_2_·6H_2_O and NaOH were purchased from Sinopharm Chemical Reagent Co., Ltd., Shanghai, China which were used as strontium source and pH regulator, respectively. All raw materials were analytically pure reagents. The raw materials with ABO_3_ atomic ratio were mixed. The A-site was strontium, and the B-site was designed to contain five equimolar elements according to Sr(Ti_0.2_Zr_0.2_Hf_0.2_V_0.2_Fe_0.2_)O_3_ and Sr(Ti_0.2_Zr_0.2_Hf_0.2_V_0.2_Nb_0.2_)O_3_. The powder was mixed with deionized water in hydrothermal synthesis reactor and stirring for 10 min to form a suspension. Then, the liquid TiCl_4_ was dropwise added into the suspension. Then, 3 mol/L NaOH solution was employed to keep the suspension a high alkaline environment pH = 11. The mixture was then transferred to a homogeneous reactor and heated at 180 °C for 12 h at 20 r/min. After the reaction, the sediment removed from the reactor was repeatedly and alternately cleaned with ethanol and deionized water by centrifugation and suction filtration until the pH value of filtrate reached 7. The high-entropy ceramic powder samples were obtained after drying the wet powder. To investigate the change of element valence state, the powder samples were calcined at different temperatures with a heating rate of 5 °C/min for 12 h. The photographs of the hydrothermal and calcined high-entropy samples are shown in Figure S1 in Supplementary Materials.

For the preparation of high-entropy ceramic powder by solid-state reaction process, submicron analytical reagents TiO_2_, ZrO_2_, HfO_2_, V_2_O_3_, Nb_2_O_5_ and SrCO_3_ were used as raw materials which were purchased from Sinopharm Chemical Reagent Co., Ltd., China. The mixed powder with Sr(Ti_0.2_Zr_0.2_Hf_0.2_V_0.2_Nb_0.2_)O_3_ atomic ratio was ball-milled with ethanol for 8 h. The mass ratio of the grinding media to the mixed powder was 2:1. The obtained powder was dried at 80 °C for 24 h, and then reacted at 1200 °C in air for 2 h with a heating rate of 5 °C/min.

### 2.2. Characterization

X-ray diffraction (XRD) using Cu K α radiation (X’Pert PRO MPD, PANalytical B⋅V., Almelo, The Netherlands) was utilized for characterizing the phase composition of perovskite powder specimens. The micromorphology of high-entropy powder was observed using Scanning electron microscope (SEM; MERLIN VP Compact, Carl Zeiss, Jena, Germany). Thermoelectric field emission multi-purpose transmission electron microscope (JEM-F200, JEOL Ltd., Akishima, Japan) with Oxford EDS detector techniques was employed to observe the microstructures of the high-entropy perovskite powder. An X-ray photoelectron spectroscopy (XPS) microprobe using a monochromated micro-focused Al Kα radiographic source with a spot size of 200–800 μm (ESCALAB 250Xi, Thermo Fisher Scientific, Waltham, MA, USA) was employed to analyze the change in the valence state at different temperatures. The absorbance of the specimens was characterized using a UV–VIS–NIR spectrophotometer (Cary 7000, Agilent, Santa Clara, CA, USA).

## 3. Results and Discussion

**Figure 1:**
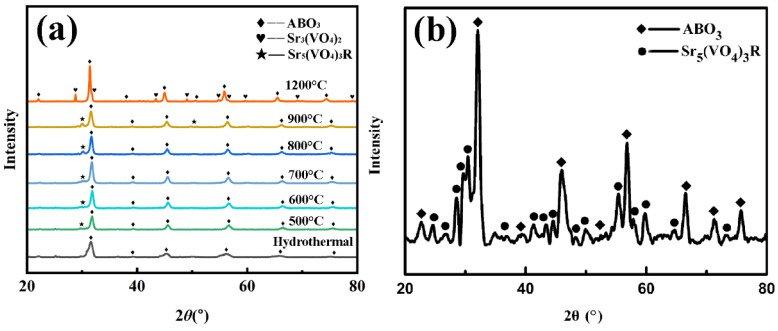
XRD spectra of high-entropy Sr(TiZrHfVNb)O_3_ system: (**a**) hydrothermal and calcined at different temperatures, (**b**) solid-state reaction.

The hydrothermal process is a common method used for the synthesis of inorganic perovskite materials. However, it is rarely used in the preparation of high-entropy ceramic powders. Most high-entropy powders are still prepared using a solid-state reaction. In this study, we synthesized the high-entropy system Sr(TiZrHfVNb)O_3_ using the hydrothermal process and calcined the materials at different temperatures. A system with the same composition was also prepared using the solid-state reaction at 1200 °C. The XRD spectra of the high-entropy samples are shown in Figure 1. It can be seen in Figure 1a that the hydrothermal sample obtained a good perovskite structure. Then, an insignificant second phase Sr_5_(VO_4_)_3_R with an apatite structure appeared in the samples calcined up to 500 °C, and stood out at 800 °C. The second phase significantly transfers to Sr_3_(VO_4_)_2_ with a commonly sintering temperature of about 1150 °C when the temperature reaches 1200 °C. It seems that Sr_5_(VO_4_)_3_R decomposes at 1200 °C. However, the second phase of the solid-state reaction sample is Sr_5_(VO_4_)_3_R at the same temperature 1200 °C, which is shown in Figure 1b. The XRD results indicate that the high-entropy Sr(TiZrHfVNb)O_3_ system can only be obtained using hydrothermal synthesis, rather than the solid-state reaction method. Thus, the hydrothermal process reveals a remarkable advantage. And, we believe that the formation of high-entropy structures is closely related to the stability of the valence states of the elements.

**Figure 2:**
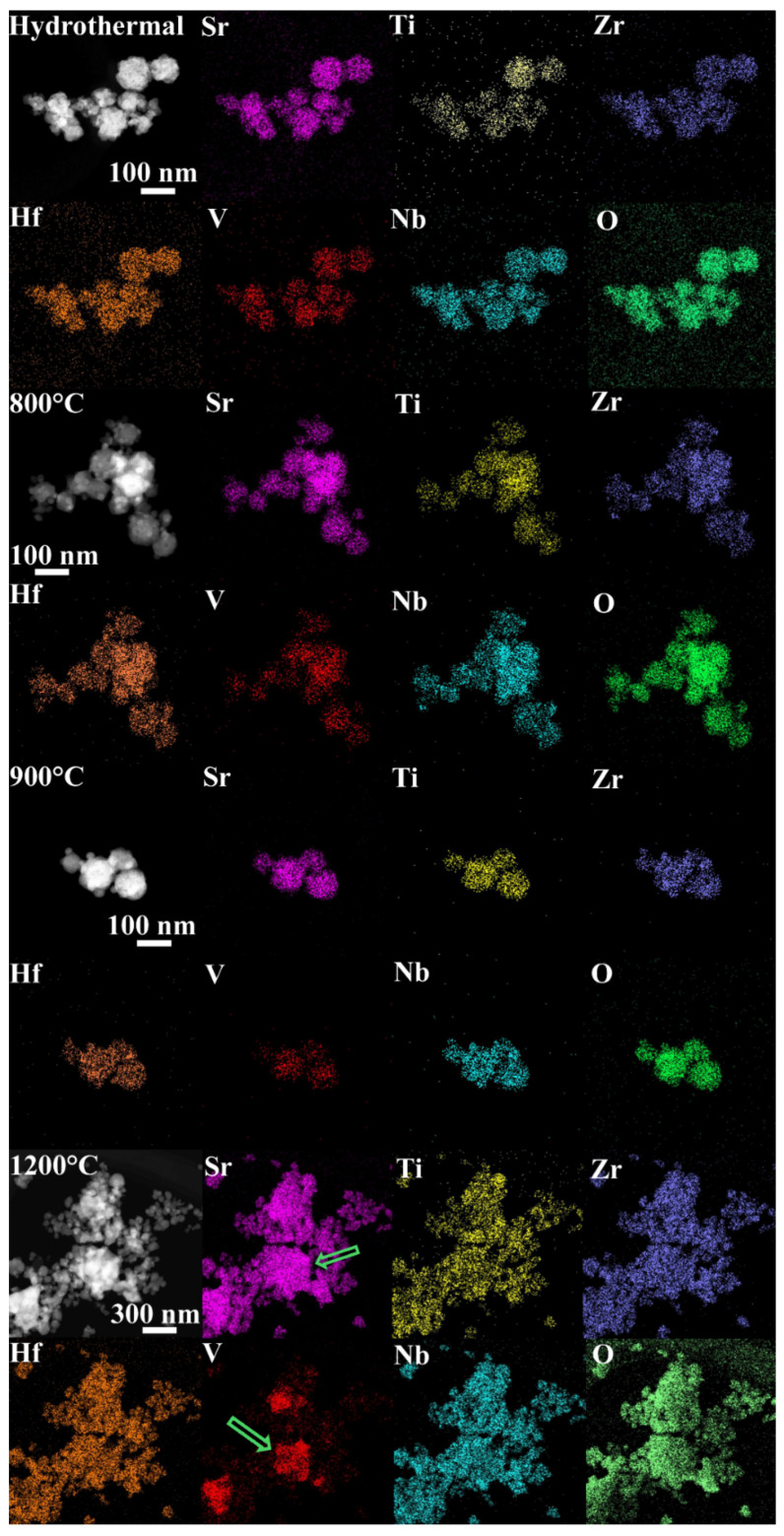
Micromorphology of Sr(TiZrHfVNb)O_3_ with EDS synthesized using hydrothermal and calcined at different temperatures.

Figure 2 shows the micromorphology of the high-entropy Sr(TiZrHfVNb)O_3_ samples with the EDS. The homogeneously distributed elements can be seen clearly in the hydrothermal and calcined 500~700 °C samples (Figures S2–S4). And, the strength of the vanadium element was weakened very slightly at 800~900 °C. Then, significant vanadium segregation appears at 1200 °C, and the strontium and oxygen elements are also clearly present in the segregation position. This is roughly consistent with the XRD results, which show a large amount of phase separation at 1200 °C. Moreover, the above phase separation phenomenon is more obviously observed in the samples prepared using the solid-state reaction (Figure S5). This indicates that the vanadium precipitates from the perovskite lattice and is combined with a portion of strontium. Furthermore, the other elements are still homogeneously distributed in the same position. This proves that other elements can still form perovskite crystals, even without the involvement of vanadium.

**Figure 3:**
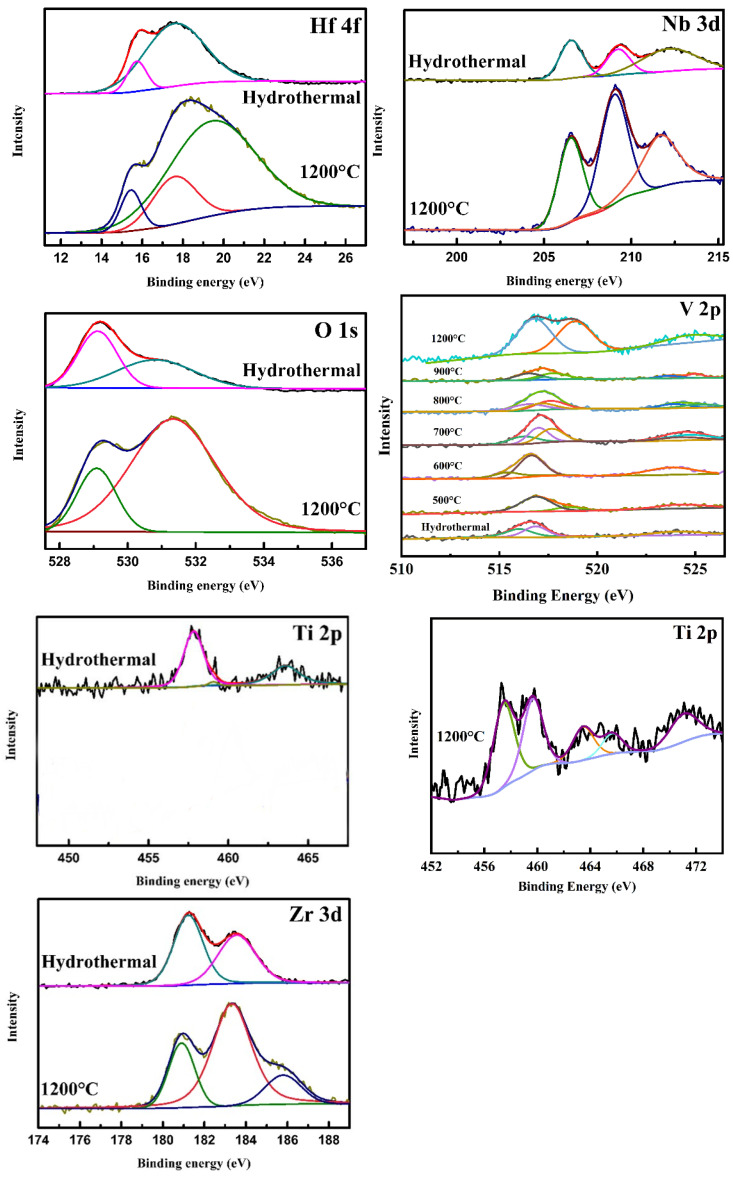
Fitted core level spectrum of the high-entropy elements in the Sr(TiZrHfVNb)O_3_ system.

In order to explore the reason for the vanadium segregation, the valence states of the designed high-entropy elements are systematically analyzed using XPS. The fitted core level spectrums of Ti2p, Zr3d, Hf4f, Nb3d and O1s from the high-entropy samples before and after calcining at 1200 °C are shown in Figure 3. The assignment of the XPS peaks of these elements is listed in Table 1. It can be seen that tetravalent and pentavalent elements keep their initial valence states in the hydrothermal samples. However, all the tetravalent elements Ti, Zr and Hf with a small percentage of trivalent peaks were detected in the high-entropy sample calcined at 1200 °C. The Nb remains constant at pentavalence. Moreover, the original percentage balance of the lattice oxygen and the vacancy oxygen is broken. The concentration of oxygen defects increased to 81.2%. The fitted core level spectrums of different elements calcined at 500~900 °C are shown in Figures S6–S10. Also, the fitted core level spectrums of V2p from the high-entropy samples before and after calcining at different temperatures are shown in Figure 3. The assignment of XPS peaks of V2p is listed in Table 2. It is clear that the vanadium(III) trichloride (VCl_3_) is used as a vanadium source in the synthesis process. The valence state of the vanadium element almost remains unchanged after the hydrothermal process. Most importantly, the percentage of V^3+^ gradually decreases from 60.9% (500 °C), 49.3% (600 °C), 25.7% (700 °C), 20.2% (800 °C) to 6.9% (900 °C), and then, all V^3+^ ascends to V^4+^ and V^5+^ at 1200 °C. The fundamental reason for the phase separation of the calcined samples is that the oxygen partial pressure is high enough to oxidize the V element. This indicates that vanadium will be gradually oxidized to a higher stable valence state with an increase in temperature. The valence stability of the vanadium leads to the valence instability of other high-entropy atoms in the perovskite lattice. That is to say, in order to stabilize the perovskite lattice with Nb^5+^, a part of the tetravalent elements is forced to be loaded with electrons from the oxygen. Thereby, the valence state of the tetravalent elements decreases, and the oxygen vacancy increases greatly. Therefore, we believe that in order to achieve the entropy stability of the perovskite lattice, it is important and necessary to maintain the valence equilibrium of the high-entropy system.

**Figure 4:**
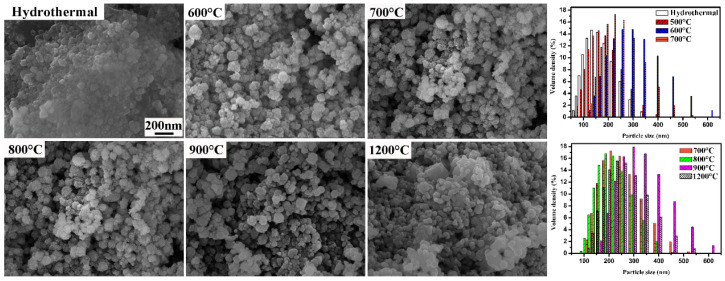
SEM images and particle size distribution of the Sr(TiZrHfVNb)O_3_ samples synthesized at different temperatures.

Figure 4 shows the microstructure and particle size distribution of the Sr(TiZrHfVNb)O_3_ samples synthesized at different temperatures. It can be observed that the average particle size of the hydrothermal samples is about 150 nm with a concentrated distribution. The particle size gradually increases when the temperature increases. After the temperature exceeds 900 °C, the particle distribution becomes more dispersed. In general, the particles are clustered in the range of 100~500 nm.

**Figure 5:**
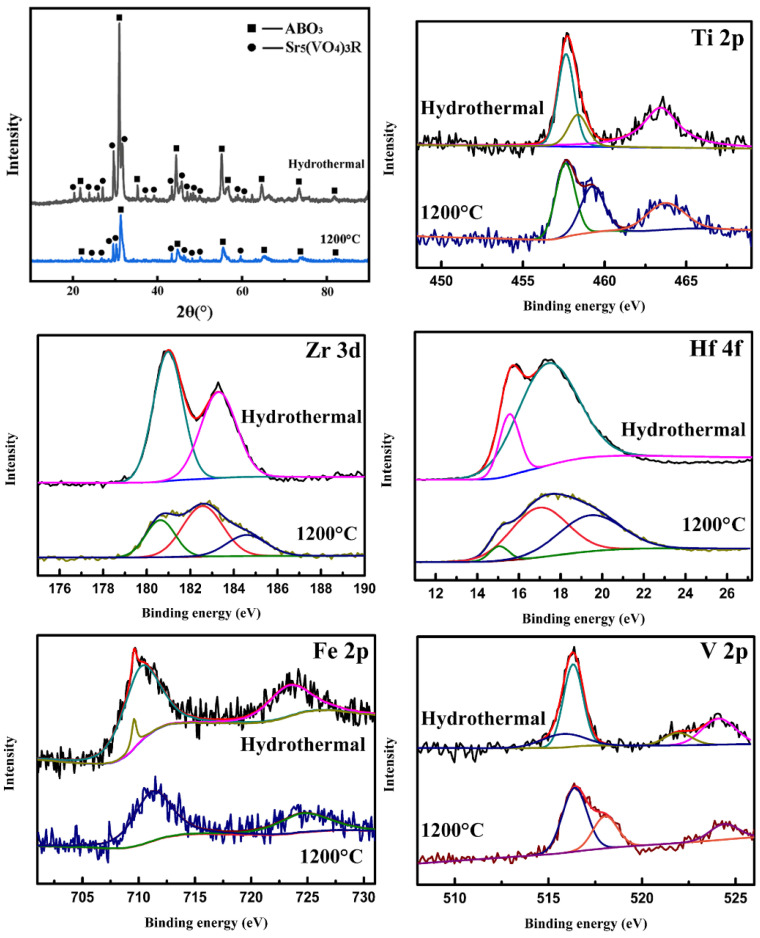
XRD spectra of the high-entropy Sr(TiZrHfVFe)O_3_ and fitted core level spectrum of the elements in this system.

To prove the above view, a high-entropy system Sr(TiZrHfVFe)O_3_ with an obviously unbalanced valence state is also designed and attempted to be synthesized using a hydrothermal process and calcined at 1200 °C. The XRD spectra of the high-entropy Sr(TiZrHfVFe)O_3_ and the fitted core level spectrum of the elements in this system are shown in Figure 5. The assignment of XPS peaks of high-entropy elements is listed in Table 3. It can be seen clearly that both the hydrothermal and the calcined samples are not pure perovskite. For the hydrothermal sample, the phase separation is easy to understand. There are no pentavalent elements in the system during the hydrothermal process. Excess trivalent elements reduce the supply of electrons. The experiment temperature also failed to provide enough energy to snatch electrons from the elements’ more stable orbits. There are too many holes without electron filling and the trivalent elements are squeezed out. In other words, the entropy value of a five-element high-entropy system is not enough to stabilize the lattice. However, the 1200 °C calcined sample should be in a balanced valence state. The single phase is still unable to be achieved. This is due to the fact that the hydroxyapatite structure already formed around the hydrothermal process is stable. It will not decompose at high temperatures to release pentavalent vanadium and refill into the perovskite lattice. Meanwhile, we believe that valence equilibrium is not the only condition to realize the entropy stability of the phase; the electronic structures of the elements must also match each other. This work is ongoing and will be published in the near future.

**Figure 6:**
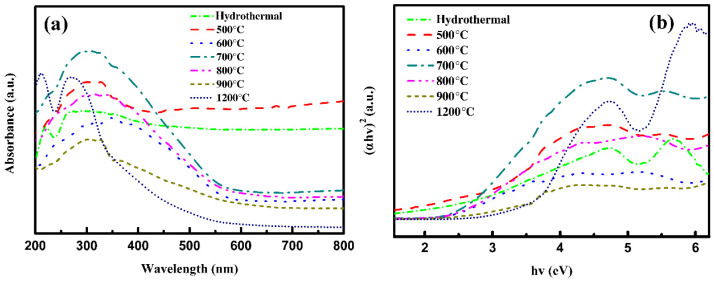
UV-Vis spectra (**a**) and the (αhv)^2^~hv curves (**b**) of Sr(TiZrHfVNb)O_3_ system.

**Figure 7:**
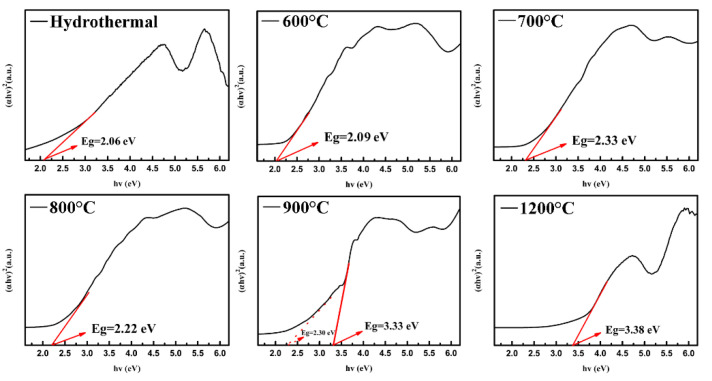
Band gap of high-entropy Sr(TiZrHfVNb)O_3_ systems synthesized using hydrothermal process and calcined different temperatures from extrapolating (αhv)^2^~hv curves.

To investigate the photoresponsiveness of the high-entropy Sr(TiZrHfVNb)O_3_ system, UV–Vis spectra and the (αhv)^2^~hv curves of the samples synthesized at different temperatures are shown in Figure 6. It can be seen that all the samples can absorb light in the ultraviolet wavelength range. However, only the hydrothermal and 500 °C calcined samples are well absorbent to the visible light wave range. The absorption of the visible range rapidly decreases with an increase in temperature, and the absorption peak of the UV range narrows gradually. Obviously, the 1200 °C calcined sample is signally absorbent to the UV range and has almost no absorption in the visible range. This is similar to most inorganic perovskites such as SrTiO_3_, BaTiO_3_, SrZrO_3_, PbTiO_3,_ and so on [18]. The band gap of the samples synthesized using hydrothermal and calcined from extrapolating (αhv)^2^~hv curves are shown in Figure 7. The hydrothermal sample has a narrow band gap of 2.06 eV. The variation trend of the band gap increases with the increase in temperature. Expressly, the 900 °C calcined sample seems to appear to have two band gaps. This is caused by the phase separation of the sample. Due to the segregation of the vanadium, part of the perovskite lattice is missing a vanadium ion, and the chemical formula shall be Sr(TiZrHfNb)O_3_ with a band gap of 3.33 eV. Thus, 900 °C can be seen as the transition temperature affecting photoresponsiveness. The species composition of the Sr(TiZrHfVFe)O_3_ system at different temperatures is listed in Table 4.

## 4. Conclusions

In summary, we designed a high-entropy Sr(TiZrHfVNb)O_3_ system, and successfully synthesized a ceramic powder with a perovskite structure using a vanadium(III) source. This system cannot be prepared using calcined or solid-state reactions in air due to the valence change of the vanadium ion. Significant phase separation and element segregation appeared in the calcined and solid phase reacted samples. The ionic valence of the vanadium ascends from V^3+^ to V^4+^ and V^5+^ with an increasing calcine temperature. The precipitated V ionic combines with a part of the Sr. It was proved that the high-entropy value could not only stabilize the crystal phase but also stabilize the ionic valence state. Also, a Sr(TiZrHfVFe)O_3_ system cannot be synthesized using the hydrothermal method. It is proposed that, for the synthesis of high-entropy perovskite, the design of entropy components to maintain the valence equilibrium is an important factor in achieving entropy stability of the phase structure. The valence state of the raw material should be carefully selected for the variable valence element sources. The ionic source valence state could be maintained during the hydrothermal process. The high-entropy Sr(TiZrHfVNb)O_3_ ceramic powder showed a wide absorption in the visible range. The high-entropy system with the vanadium element might achieve a promising material for optical applications.

## Figures and Tables

**Table 1:** Assignment of XPS peaks of different elements in Sr(TiZrHfVNb)O_3_ system before and after calcining at 1200 °C.

Element	Binding Energy (eV)		Percentage (%)		Species	
	180 °C	1200 °C	180 °C	1200 °C	180 °C	1200 °C
Ti 2p	457.8	457.46	62.9	41.1	2p3/2 RTiO_3_	2p3/2 Ti^3+^
	459.1	459.65	1.8	33.1	2p3/2 TiO_2_	2p3/2 Ti^4+^
	463.6	463.46	35.3	16.4	2p1/2 TiO_2_	2p1/2 Ti^3+^
	-	465.65	-	9.4	-	2p1/2 Ti^4+^
Zr 3d	181.4	180.9	54.0	22.7	3d5/2 ZrO_2_	3d5/2 ZrO_2_/Zr^3+^
	183.6	183.3	46.0	63.3	3d5/2 ZrO_2_	3d5/2 ZrO_2_
	-	185.8	-	14.0	-	3d3/2 ZrO_2_
Hf 4f	16.0	15.4	13.3	7.9	4f7/2 HfO_2_	4f7/2 Hf^3+^
	17.6	17.6	86.7	18.7	4f5/2 HfO_2_	4f5/2 HfO_2_
	-	19.4	-	73.4	-	4f5/2 HfO_2_
Nb 3d	206.5	206.5	32.4	28.3	3d5/2 ANbO_3_/Nb_2_O_5_	3d5/2 ANbO_3_/Nb_2_O_5_
	209.2	209.0	22.4	41.7	3d3/2 ANbO_3_	3d3/2 ANbO_3_
	212.2	211.7	45.2	30.0	3d3/2 Nb_2_O_5_	3d3/2 Nb_2_O_5_
O 1s	529.1	529.1	50.6	18.8	lattice oxygen	
	530.8	531.3	49.4	81.2	vacancy oxygen	

**Table 2:** Assignment of XPS peaks of V2p in Sr(TiZrHfVNb)O_3_ system before and after calcining at different temperatures.

Temperature ( °C)	Binding Energy (eV)	Percentage (%)	Species
Hydrothermal	515.8	38.5	3/2 V_2_O_3_
	516.8	36.3	3/2 V_2_O_3_
	524.0	25.2	1/2 V^3+^
500	516.9	60.9	3/2 V_2_O_3_
	518.6	14.1	3/2 V_2_O_5_
	524.3	25.0	1/2 VO_2_/V_2_O_5_
600	516.2	18.9	3/2 VO_2_
	517.2	49.3	3/2 V_2_O_3_
	524.3	31.8	1/2 VO_2_/V_2_O_5_
700	516.2	17.9	3/2 VO_2_
	516.9	25.7	3/2 V_2_O_3_
	517.6	26.5	3/2 V_2_O_5_
	524.2	10.5	1/2 VO_2_
	524.6	19.4	1/2 V_2_O_5_
800	516.4	23.4	3/2 VO_2_
	517.2	20.2	3/2 V_2_O_3_
	517.6	29.1	3/2 V_2_O_5_
	523.9	13.6	1/2 VO_2_
	525.2	13.7	1/2 V_2_O_5_
900	516.4	32.6	3/2 VO_2_
	517.2	6.9	3/2 V_2_O_3_
	517.5	32.9	3/2 V_2_O_5_
	523.5	10.8	1/2 VO_2_
	524.9	16.8	1/2 V_2_O_5_
1200	516.5	45.1	3/2 VO_2_
	518.6	40.8	3/2 V_2_O_5_
	524.9	14.1	1/2 VO_2_/V_2_O_5_

**Table 3:** Assignment of XPS peaks of different elements in Sr(TiZrHfVFe)O_3_ system.

Element	Binding Energy (eV)		Percentage (%)		Species	
	180 °C	1200 °C	180 °C	1200 °C	180 °C	1200 °C
Ti 2p	457.7	457.6	40.8	40.9	2p3/2 RTiO_3_	2p3/2 RTiO_3_
	458.5	459.3	10.2	30.7	2p3/2 TiO_2_	2p3/2 TiO_2_
	463.4	463.7	50.0	28.4	2p1/2 Ti^4+^	2p1/2 Ti^4+^
Zr 3d	180.9	180.6	55.3	30.9	3d5/2 ZrO_2_/Zr^3+^	3d5/2 Zr^3+^
	183.3	182.6	44.7	57.3	3d5/2 ZrO_2_	3d5/2ZrO_2_
	-	184.6	-	11.8	-	3d3/2ZrO_2_
Hf 4f	15.5	15.1	17.6	5.5	4f7/2 Hf^3+^	4f7/2 Hf^3+^
	17.3	17.3	82.4	52.2	4f5/2 HfO_2_	4f5/2 HfO_2_
	-	19.8	-	42.3	-	4f5/2 HfO_2_
Fe 2p	710.2	711.2	64.5	65.4	2p3/2 Fe_3_O_4_	2p3/2 Fe_2_O_3_
	709.6	724.6	4.3	34.6	2p3/2 FeO	2p1/2 Fe_2_O_3_
	723.3	-	31.2	-	2p1/2 Fe_3_O_4_	
V 2p	515.8	516.4	16.7	56.1	3/2 V_2_O_3_	3/2 VO_2_
	516.3	518.0	47.6	28.0	3/2 VO_2_	3/2 V_2_O_5_
	521.9	524.4	10.2	15.9	1/2 V^3+^	1/2 VO_2_/V_2_O_5_
	524.1		25.5		1/2 VO_2_	

**Table 4:** Species composition of the Sr(TiZrHfVFe)O_3_ system at different temperatures.

Species	Hydrothermal	500~800 °C	900 °C	1200 °C
Sr(TiZrHfVNb)O_3_	√	-	-	-
Sr(TiZrHfV_0.2−x_Nb)O_3_	-	√	√	-
Sr(TiZrHfNb)O_3_	-	-	√	√
Sr_5_(VO_4_)_3_R	-	√	√	-
Sr_3_(VO_4_)_2_	-	-	-	√

## Data Availability

The original contributions presented in the study are included in the article and Supplementary Materials, further inquiries can be directed to the corresponding authors.

## Supplementary Materials

The following supporting information can be downloaded at: https://www.mdpi.com/article/10.3390/ma17174275/s1, Figure S1. Photograph of the high-entropy Sr(TiZrHfVNb)O3 samples synthesized by hydrothermal and calcined at different temperatures. Figure S2. Micromorphology of Sr(TiZrHfVNb)O3 with EDS after calcining at 500 °C. Figure S3. Micromorphology of Sr(TiZrHfVNb)O3 with EDS after calcining at 600 °C. Figure S4. Micromorphology of Sr(TiZrHfVNb)O3 with EDS after calcining at 700 °C. Figure S5. Micromorphology of Sr(TiZrHfVNb)O3 with EDS synthesized by solid-state reaction at 1200 °C. Figure S6. Fitted core level spectrum of elements in the Sr(TiZrHfVNb)O3 system after calcining at 500 °C. Figure S7. Fitted core level spectrum of elements in the Sr(TiZrHfVNb)O3 system after calcining at 600 °C. Figure S8. Fitted core level spectrum of elements in the Sr(TiZrHfVNb)O3 system after calcining at 700 °C. Figure S9. Fitted core level spectrum of Zr element in the Sr(TiZrHfVNb)O3 system after calcining at 800 °C. Figure S10. Fitted core level spectrum of elements in the Sr(TiZrHfVNb)O3 system after calcining at 900 °C. Figure S11. Fitted core level spectrum of elements in the Sr(TiZrHfVNb)O3 system prepared by solid state reaction. Figure S12. Fitted core level spectrum of the elements in Sr(TiZrHfVFe)O3 system. Figure S13. Band gap of high entropy Sr(TiZrHfVNb)O3 system after calcining at 500 °C from extrapolating (αhv)2~hv curves.
