# Supplementary material for: Hydrothermal Synthesis of a Valence State Constant High-Entropy Perovskite Sr(TiZrHfVNb)O3 with Improved Photoresponsiveness
Source: Materials (Basel). 2024 Aug 29;17(17):4275. doi: 10.3390/ma17174275 (PMC11396518; doi:10.3390/ma17174275)
Supplement: Supplementary file 1 [file materials-17-04275-s001.zip › materials-3146686-supplementary.pdf]

Supplementary Information

Hydrothermal Synthesis of a Valence State Constant High-Entropy Perovskite Sr(TiZrHfVNb)O_3 with Improved Photoresponsiveness

Yihua Bai [1], Ke Gan [2,3] *, Xiaohu Li [4], and Dongping Duan [2,3] *

[1] School of Materials Science and Engineering, University of Science and Technology Beijing, Beijing 100083, China

[2] CAS Key Laboratory of Green Process and Engineering, National Engineering Research Center of Green Recycling for Strategic Metal Resources, Institute of Process Engineering, Chinese Academy of Sciences, Beijing 100190, China

[3] University of Chinese Academy of Sciences, Beijing 100049, China

[4] Institute for Advanced Materials and Technology, University of Science and Technology Beijing, Beijing 100083, China

*Corresponding authors.

Tel./Fax: +86-10-82544874

E–mail: jdzganke@163.com, douglass@ipe.ac.cn

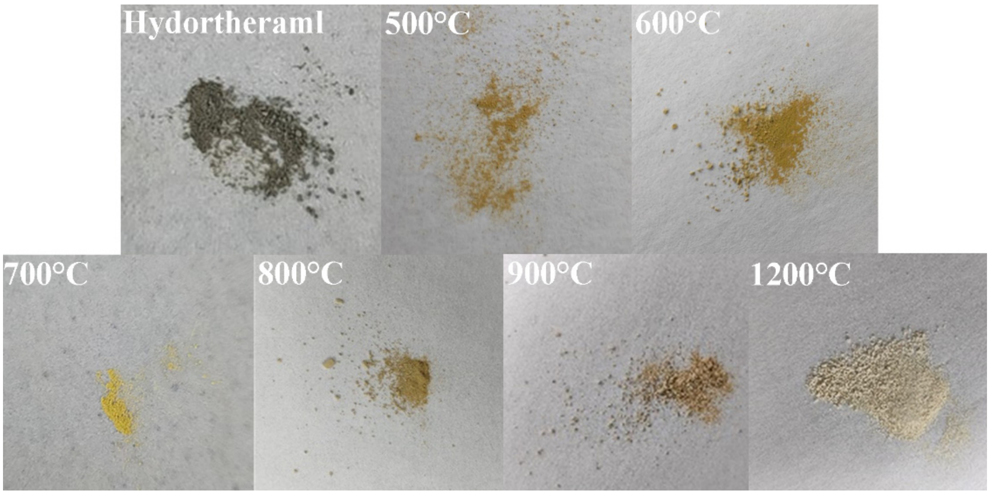

Figure. S1 Photograph of the high-entropy $Sr(TiZrHfVNb)O_3$ samples synthesized by hydrothermal and calcined at different temperatures

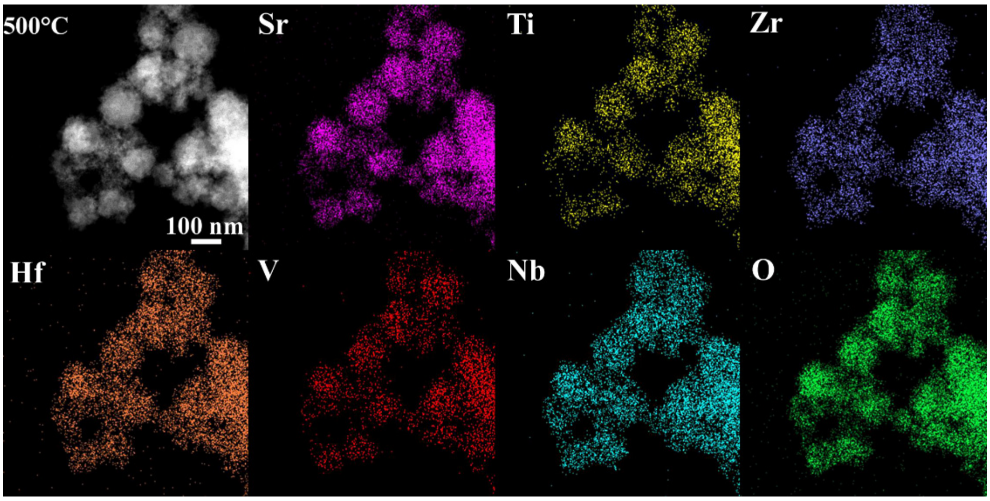

Figure. S2 Micromorphology of $Sr(TiZrHfVNb)O_3$ with EDS after calcining at 500°C

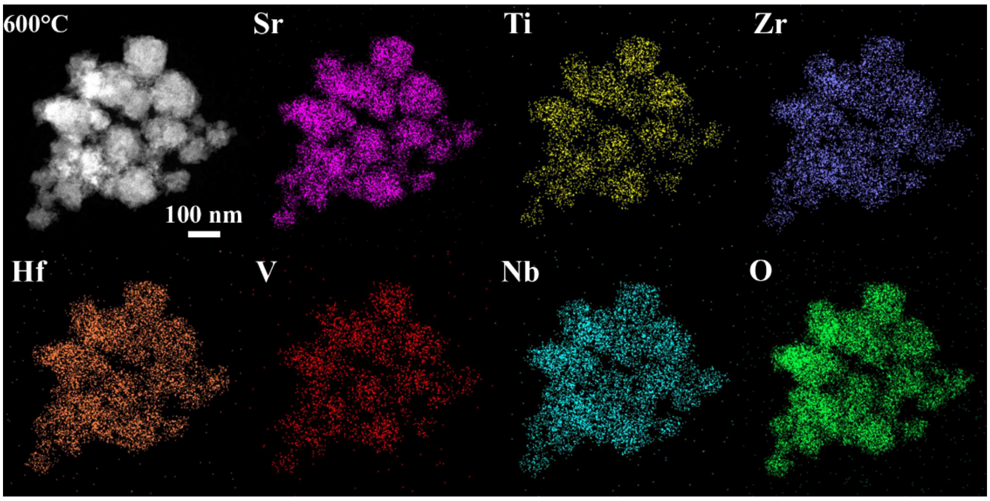

Figure. S3 Micromorphology of Sr(TiZrHfVNb)O_3 with EDS after calcining at 600°C

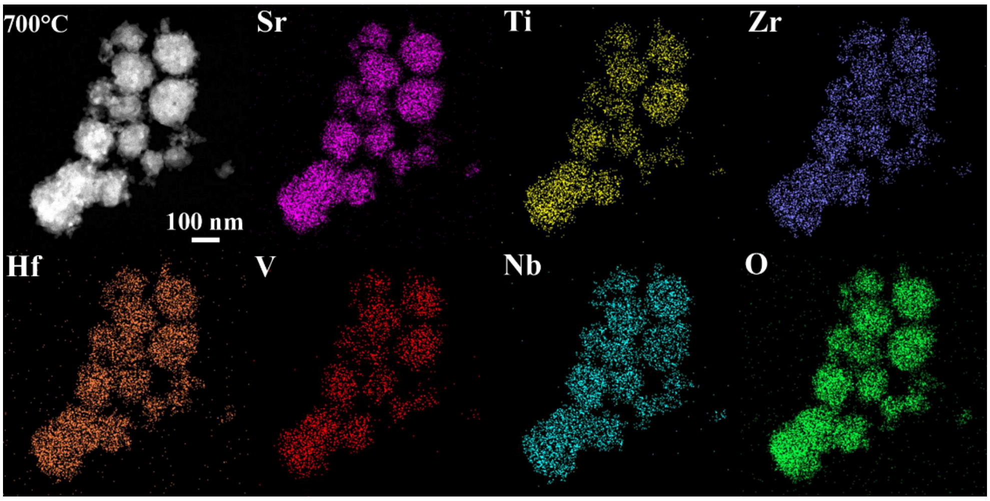

Figure. S4 Micromorphology of Sr(TiZrHfVNb)O_3 with EDS after calcining at 700°C

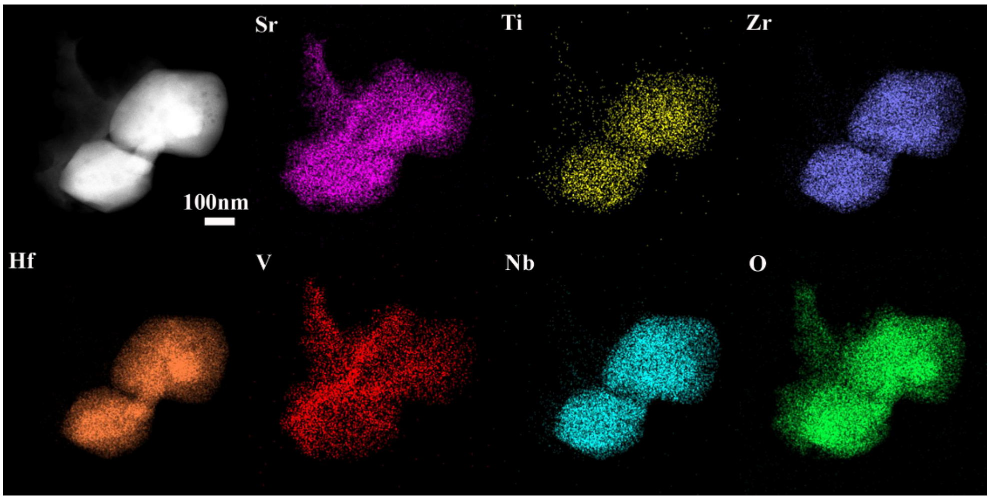

Figure. S5 Micromorphology of Sr(TiZrHfVNb)O_3 with EDS synthesized by solid-state reaction at 1200°C

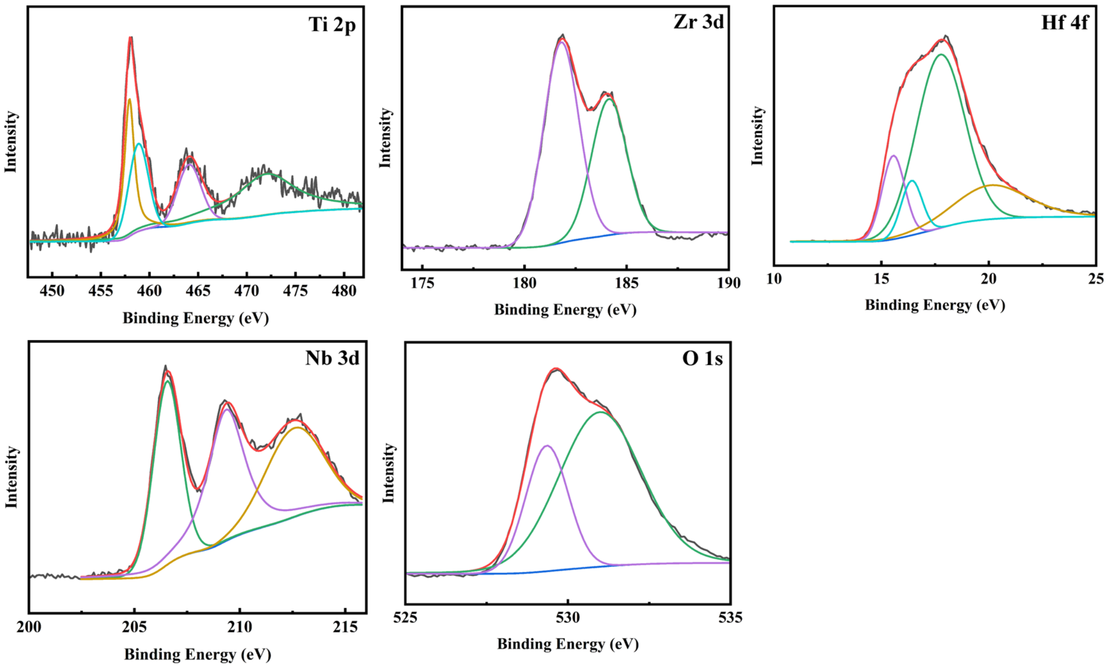

Figure. S6 Fitted core level spectrum of elements in the Sr(TiZrHfVNb)O_3 system after calcining at 500°C.

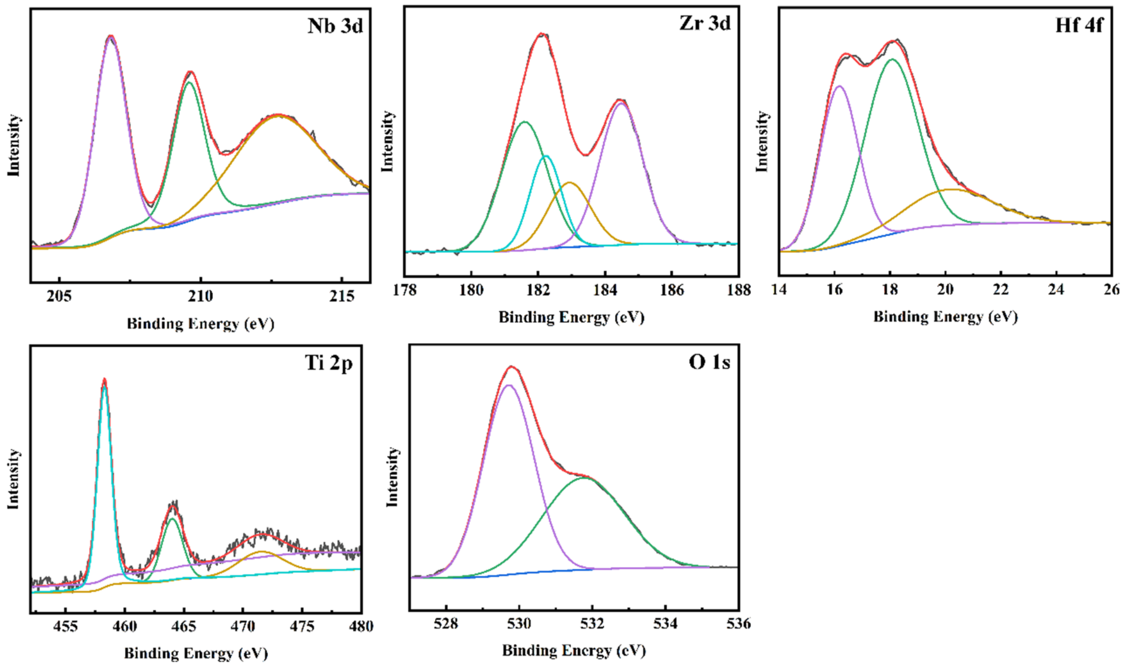

Figure. S7 Fitted core level spectrum of elements in the Sr(TiZrHfVNb)O_3 system after calcining at 600°C.

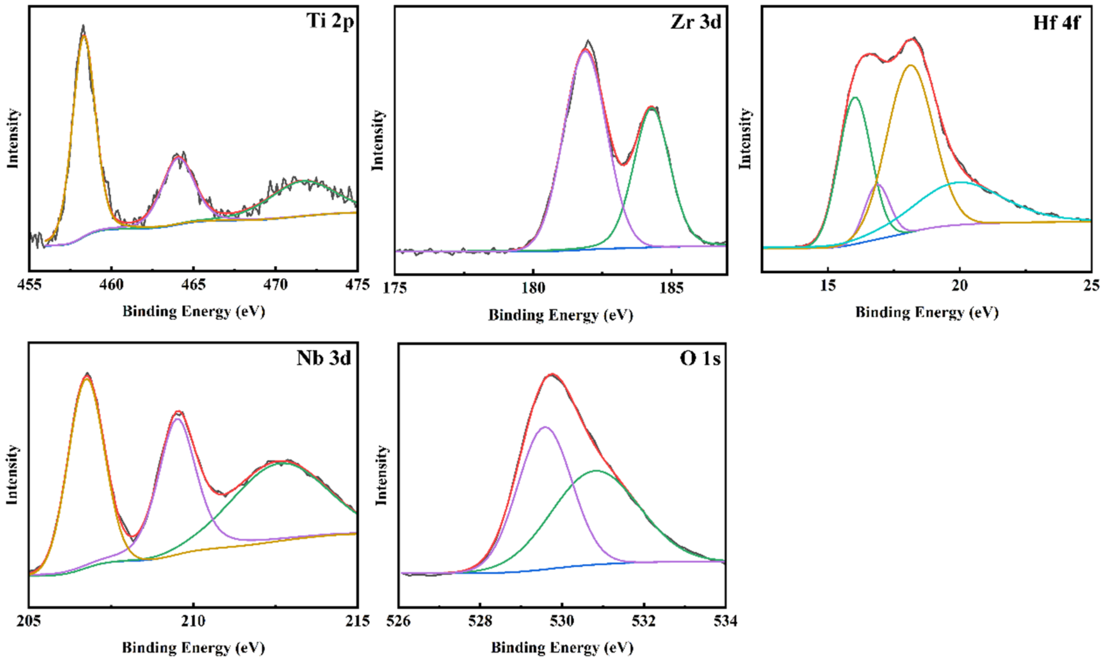

Figure. S8 Fitted core level spectrum of elements in the Sr(TiZrHfVNb)O_3 system after calcining at 700°C.

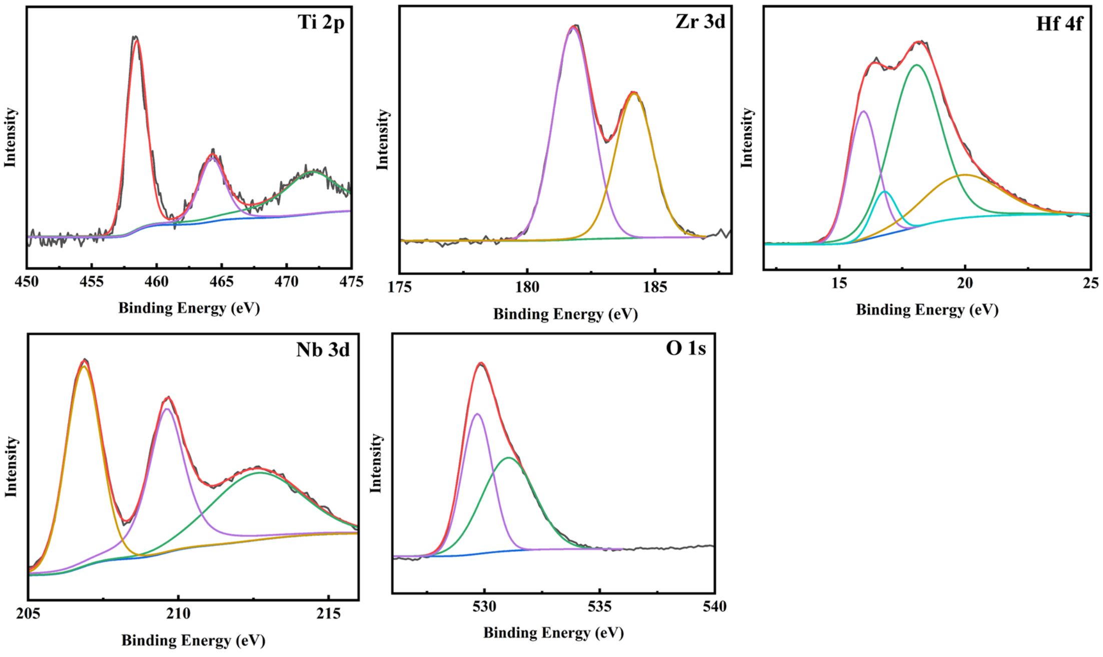

Figure. S9 Fitted core level spectrum of Zr element in the Sr(TiZrHfVNb)O_3 system after calcining at 800°C.

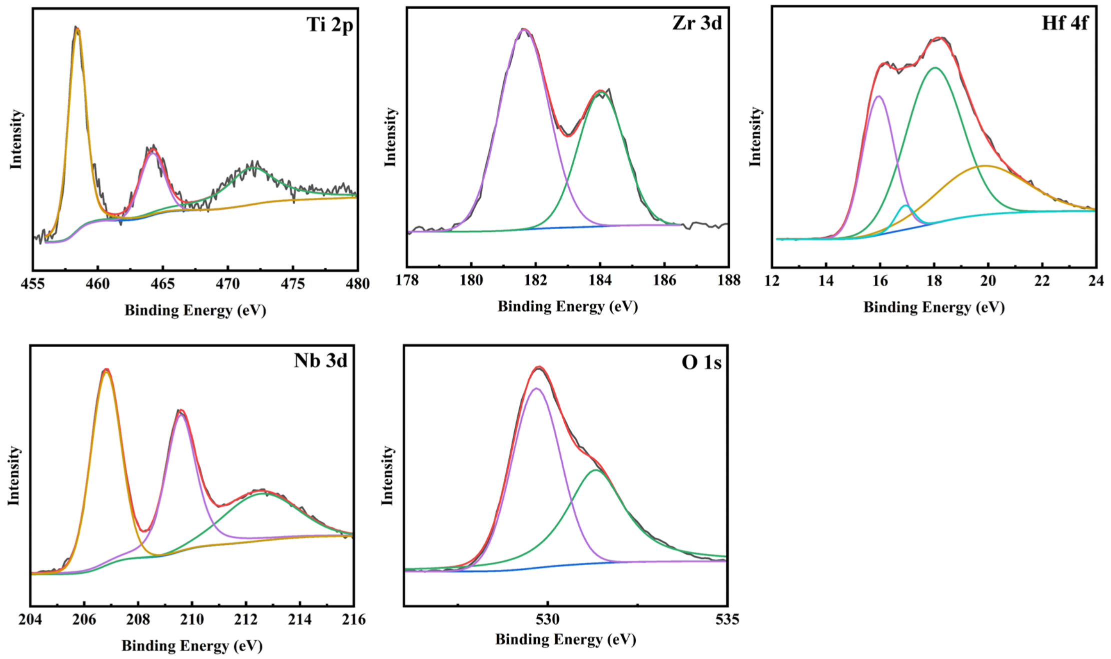

Figure. S10 Fitted core level spectrum of elements in the Sr(TiZrHfVNb)O_3 system after calcining at 900°C.

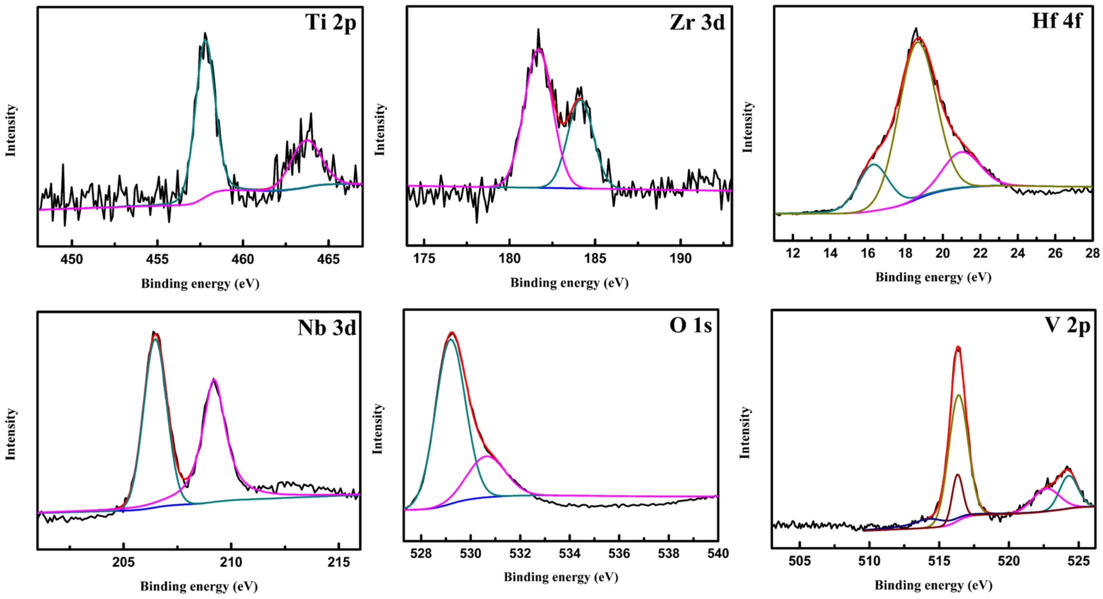

Figure. S11 Fitted core level spectrum of elements in the Sr(TiZrHfVNb)O_3 system prepared by solid state reaction.

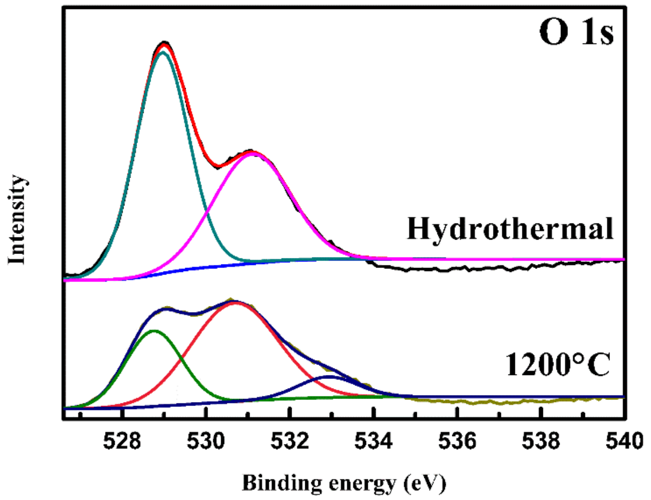

Figure. S12 Fitted core level spectrum of the elements in Sr(TiZrHfVFe)O_3 system.

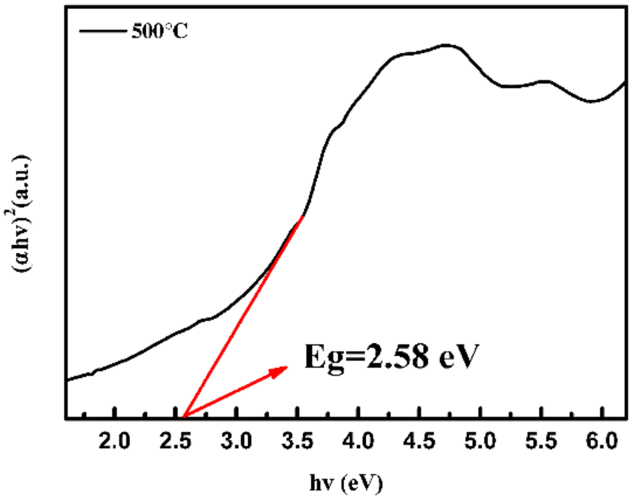

Figure. S13 Band gap of high entropy Sr(TiZrHfVNb)O_3 system after calcining at 500°C from extrapolating $(\alpha hv)^2$~hv curves.
